# A subcritical water extract of soil grown *Zingiber officinale* Roscoe: Comparative analysis of antioxidant and anti-inflammatory effects and evaluation of bioactive metabolites

**DOI:** 10.3389/fphar.2023.1006265

**Published:** 2023-02-08

**Authors:** Azraul Mumtazah Razak, Siti Nor Asyikin Zakaria, Nur Fathiah Abdul Sani, Nazirah Ab Rani, Nur Haleeda Hakimi, Mazlina Mohd Said, Jen Kit Tan, Han Kwang Gan, Mariam Firdhaus Mad Nordin, Suzana Makpol

**Affiliations:** ^1^ Department of Biochemistry, Faculty of Medicine, Universiti Kebangsaan Malaysia, Kuala Lumpur, Malaysia; ^2^ Faculty of Health Sciences, University College of MAIWP International, Kuala Lumpur, Malaysia; ^3^ Centre of Drug and Herbal Research, Faculty of Pharmacy, Universiti Kebangsaan Malaysia, Kuala Lumpur, Malaysia; ^4^ Millercle Resources Sdn Bhd, Klang, Malaysia; ^5^ AM Zaideen Ventures Sdn Bhd, Kuala Lumpur, Malaysia

**Keywords:** *Zingiber officinale* Roscoe, antioxidant, anti-inflammatory, anti-ageing, sub-critical water extraction

## Abstract

**Introduction:** Ginger (*Zingiber officinale* Roscoe) can scavenge free radicals, which cause oxidative damage and inflamm-ageing. This study aimed to evaluate the antioxidant and anti-inflammatory effects of soil ginger's sub-critical water extracts (SWE) on different ages of Sprague Dawley (SD) rats. The antioxidant properties and yield of SWE of soil- and soilless-grown ginger (soil ginger and soilless ginger will be used throughout the passage) were compared and evaluated.

**Methods:** Three (young), nine (adult), and twenty-one (old) months old SD rats were subjected to oral gavage treatments with either distilled water or the SWE of soil ginger at a concentration of 200 mg/kg body weight (BW) for three months.

**Results:** Soil ginger was found to yield 46% more extract than soilless ginger. While [6]-shogaol was more prevalent in soilless ginger, and [6]-gingerol concentration was higher in soil ginger (*p* < 0.05). Interestingly, soil ginger exhibited higher antioxidant activities than soilless ginger by using 2,2-diphenyl-1-(2,4,6-trinitrophenyl) hydrazyl (DPPH) and ferric reducing antioxidant power (FRAP) assay. With ginger treatment, a reduced levels of tumour necrosis factor-α (TNF-α) and C-reactive protein (CRP) but not interleukin-6 (IL-6) were observed in young rats. In all ages of SD rats, ginger treatment boosted catalase activity while lowering malondialdehyde (MDA). Reduction of urine 15-isoprostane F_2t_ in young rats, creatine kinase-MM (CK-MM) in adult and old rats and lipid peroxidation (LPO) in young and adult rats were also observed.

**Discussion:** The findings confirmed that the SWE of both soil and soilless grown ginger possessed antioxidant activities. Soil ginger produced a higher yield of extracts with a more prominent antioxidant activity. The SWE of soil ginger treatment on the different ages of SD rats ameliorates oxidative stress and inflammation responses. This could serve as the basis for developing a nutraceutical that can be used as a therapeutic intervention for ageing-related diseases.

## 1 Introduction

Ginger is a Zingiberaceae perennial plant. It has been harvested for countless generations as a spice and use in herbal remedies (Mohd et al., 2012). Ginger can be collected whether it is young (between three and 4 months old) or mature (8–10 months) (Marsh et al., 2021). Nutritional support for the growth of a plant is provided by soil (Fussy and Papenbrock, 2022). The conventional method of ginger cultivation is soil-bound, using a shifting cultivation technique. This technique mainly produces land corrosion in the highlands, and it takes 6 years to resolve the issue of soil infertility before replanting can be done (Shuhaimi et al., 2016). Consequently, a different approach to solving this issue is to grow ginger using the soilless culture system. By growing ginger without soil, soilborne ailments like *Fusarium oxysporum, Pseudomonas solanacearum* that might infect the plant root, and leaf spot illnesses, can be avoided. The availability of water and nutrients for the plant may be significantly influenced by the substrate, which may also impact on the metabolic processes involved in synthesing the bioactive chemicals. This technique uses substrates of rock wool, perlite, vermiculite and peat (Mohd et al., 2015).

Several bioactive compounds are present in ginger. They include terpenes (Halimin et al., 2022) and phenolic substances such as gingerols, shogaols, and paradols. The combinations of [6]-gingerol, [6]-shogaol, [10]-gingerol, gingerdiones, gingerdiols, paradols, [6]-dehydrogingerols, [5]-acetoxy-6-gingerol, [3,5]-diacetoxy-[6]-gingerdiol, and [12]-gingerol are responsible for its recognised biological activity (Zammel et al., 2021; Arcusa et al., 2022). The two most potent active ingredients are [6]-shogaol and [6]-gingerol (Mao et al., 2019; Aghamohammadi et al., 2020; Zammel et al., 2021). Studies on ginger rhizomes have revealed that it exhibits a wide range of bioactivities including neuroprotective (Sapkota et al., 2019), hepatoprotective (Bekkouch et al., 2022), gastroprotective effects (Ebrahimzadeh Attari et al., 2019), photoprotective effect (Nobile et al., 2016), antimicrobial (Abdullahi et al., 2020), anti-obesity (Seo et al., 2021), anticancer (Osman et al., 2021), anti-inflammatory (Askari et al., 2020) and antioxidant properties (Hur et al., 2020).

A state known as “oxidative stress”, which is defined as “an imbalance between reactive species (RS), reactive oxygen species (ROS), and antioxidant reserve”, is linked to ageing and chronic disease (Mao et al., 2019). Alterations in DNA transcription and a reduction in the ability to repair DNA could result from oxidative damage to macromolecules such as lipids, proteins and DNA. Oxidative stress and high concentration of polyunsaturated fatty acids (PUFAs) in cellular or organelle membranes induce lipid peroxidation which results in the release of α - and β-unsaturated reactive aldehydes (Taso et al., 2019) such as malondialdehyde (MDA), 4-hydroxy-2-noneal (HNE), and acrolein, which are the most reactive (Mas-Bargues et al., 2021). When ROS oxidize membrane phospholipids, lipid hydroperoxide (LPO) molecules are produced within the cell membrane (Thimraj et al., 2018). These aldehydes can bond covalently with biological components. Besides, the oxidation of arachidonic acid and docosahexaenoic acid (DHA) also produces lipid peroxidation products such as isoprostanes (IsoPs) and neuroprostanes (neuroPs) (Taso et al., 2019). They also cause an increase in nitric oxide (NO) which subsequently leads to a considerable drop in the blood levels of endogenous antioxidants such as glutathione (GSH), superoxide dismutase (SOD) and catalase (CAT) (Nandi et al., 2019).

The imbalance of the normal redox state exponentially develops with age and is accompanied by a remarkable decrease in the cell repair system. Inflammageing, a modest, low-grade chronic inflammatory condition, has been reported to contribute to ageing (Sanada et al., 2018) with an elevation of proinflammatory cytokines, chemokines, and adipokines interleukin-1ß (IL-1ß), interleukin-6 (IL-6), tumour necrosis factor-α (TNF-α), and monocyte chemoattractant protein-1 chemokine (C-C motif ligand 2, CCL2) as the most important characteristics (Jalali et al., 2020). In healthy individuals, initial defence against pathogens and the injury-repair cycle depends on inflammation (Petersen and Smith, 2016). These inflammatory mediators may persist excessively for a long time, leading to chronic inflammation. Chronic inflammatory diseases can cause persistently high C-reactive protein (CRP) values (Sproston and Ashworth, 2018). The aberrant release of TNF-α causes psoriasis, psoriatic arthritis, and non-infectious uveitis (NIU) (Zhou et al., 2020; Jang et al., 2021). As ageing is the primary risk factor for most neurodegenerative diseases in humans (Hou et al., 2019), age-related disorders may be delayed, if not prevented, by therapies that use phytochemicals to target the ageing process. Since the antioxidant properties of plants could stem from their polyphenolic compounds, it is crucial to assess the polyphenolic contents of these plants, which could be extracted using various techniques.

Various extraction techniques which incorporated inorganic solvents can be used in the plant extraction process (Dias et al., 2021). The use of these solvents during the extraction and downstream processing of medicinal herbs is constrained as many organic solvents can be toxic to humans, depending on the level of exposure. It is almost impossible to remove the residual solvents completely from liquid and dried herbal extracts (Truong et al., 2019). Subcritical-water extraction, an eco-friendly process uses only water to extract phytochemical components and can concentrate less-polar chemicals quickly (Ko et al., 2020). Hence, this study aimed to evaluate the antioxidant and anti-inflammatory effects of the sub-critical water extracts of soil ginger on different ages of Sprague Dawley (SD) rats. We also assessed and compared the antioxidant properties and yield of SWE of soil- and soilless-produced ginger.

## 2 Materials and methods

### 2.1 Plant materials

Thirty kilograms of fresh rhizomes of young soil grown ginger and 30 kg of fresh rhizomes of young soilless grown ginger aged 150 days were supplied by Millercle Resources Sdn. Bhd. The soilless ginger grows in 100% cocopeat. The voucher specimens were identified and deposited at the Universiti Kebangsaan Malaysia’s Herbarium (UKMB), Bangi (ID016/2021).

### 2.2 Extraction

Unpeeled ginger rhizomes were washed and grinded to increase the surface area by 1:3 solid-to-liquid ratio. The extraction was performed in 70 L subcritical water extraction at the optimum temperature and pressure of 120°C and 10 bar, respectively. The processing time of 5, 10, 15 and 20 min were used with a solvent-to-solid ratio of 28/2 mLmg^−1^. The subcritical water extraction was performed by inserting the grounded ginger (weight varied based on the solvent-to-solid ratio for each run) into a sample holder placed in the extraction vessel and filled with distilled water (2.5 L). A fitting cover was installed on top of the extraction vessel to prevent pressure loss during the extraction process. Nitrogen gas was used to purge the oxygen present in the solution. The required pressure was maintained until the experiment was completed. Finally, the extracts were streamed into the cooling vessels to be collected for analysis and further drying.

### 2.3 Chromatographic analysis

Two selected chemical markers for the extract, [6]-gingerol and [6]-shogaol were identified and quantified using HPLC (Waters Alliance 2,695, United States) equipped with a PhotoDiode Array Detector (Waters 2,996, United States) Table 1 and the Empower Chromatography Data System for data processing. Analysis method were adapted from Mohd Sahardi et al. (2021) and optimized to the following parameters:

**TABLE 1:** HPLC parameters for chromatographic analysis.

HPLC Parameters	Condition
Column	C_18_ reversed-phase column
Mobile phase	As the mobile phases A and B, respectively, we utilized water and 100% acetonitrile (Merck, Germany). The active components of ginger were separated as follows: the volumetric ratio was 70:30 from 0 to 15 min; 5.0:95 from 15 to 16 min; 5.0:95 from 16 to 17 min; and 70:30 from 17 to 20 min.
Flow rate	0.4 mL/min
Detector	Photodiode array (PDA) at 282 nm

The extraction yield was calculated using the equation below:

$$Extraction\;yield\;\left(\%\;yield\right)=\left(W1*100\right)/W2$$

*W1 is the weight of the extract
*W2 is the weight of the plant powder

### 2.4 Preparation of controls for ferric reducing antioxidant power (FRAP) and 2,2-diphenyl-1-picrylhydrazyl (DPPH) assays

Tocotrienol-rich fraction (TRF) purchased from Sime Darby Sdn. Bhd., Selangor, Malaysia (TRF Gold Tri E 50) and Vitamin C (L-ascorbic acid 99%, Sigma, United States) were used as controls for the DPPH assay. Each ginger extract and control were weighed and diluted with either 100% ethanol or 95% ethanol and ultrapure water based on its solubility characteristics to create 10 mg/mL of the stock solutions.

### 2.5 DPPH free radical scavenging activity

The free radical scavenging activity of ginger extracts was measured using DPPH. Briefly, 40 mL of acetate buffer and 60 mL of methanol (both from Merck, Germany) were combined to make the stock solution of 1,1-diphenyl-2-picryl-hydrazyl (Sigma, United States) (pH 5.5). The appropriate diluents were used to dilute a series of final concentrations of the ginger extracts and the controls to 0, 10, 20, 50, 100, 200, 500, and 1,000 μg/mL. Then, a vortex was used to combine .75 mL of the diluted extract or control with 1.5 mL of .009 mgmL^−1^ DPPH in methanol. Following the incubation, the mixture was let to stand at room temperature for 10 min. The absorbance was determined at a wavelength of 517 nm by EnSpire Multimode Plate Reader (Perkin Elmer, Singapore). Methanol served as a standard (Ac). The inhibition percentage was calculated using the formula below (Garcia et al., 2012):

$$Inhibition=\frac{Ac-As}{Ac}\;x\;100$$

### 2.6 FRAP assay

Two point 5 mL of TPTZ 2,4,6-tri (2-pyridyl-1,3,5-triazine) (Sigma, United States), 2.5 mL of ferric chloride hexahydrate (FeCl3.6H2O) solution (20 mM) (Merck, United States), and 2.5 mL of acetate buffer (300 mM; pH 3.6) (Merck, Germany) were mixed in a ratio of 1:1:30 to create the FRAP reagent. Then, 1.2 mL of the FRAP reagent were added to the ginger extract and control solution to make a final concentration of 0, 10, 20, 50, 100, 200, 500, or 1,000 µgmL^−1^. The mixtures were incubated for 10 min and absorbance were recorded at 593 nm by EnSpire Multimode Plate Reader (Perkin Elmer, Singapore). The sample’s antioxidant capacity was assessed using a standard curve of ferrous sulfate (FeSO_4_7H_2_O) (Sigma, United States).

### 2.7 Animal model

**FIGURE 1:**
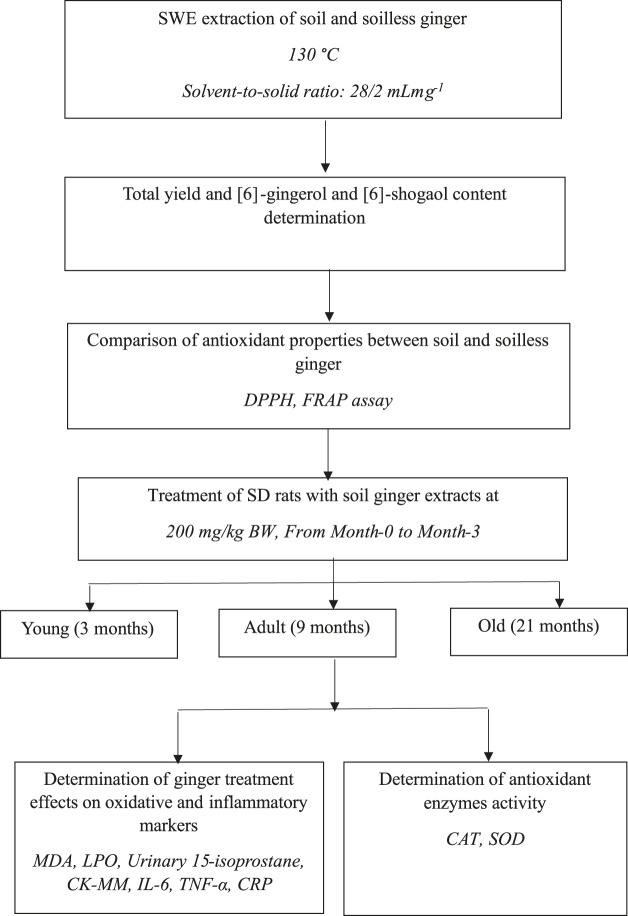
Diagram showing the experimental process and the study’s objectives.

The Sprague Dawley (SD) rats were purchased from the Universiti Kebangsaan Malaysia Laboratory Animal Resource Unit (LARU). Throughout the study, the rats were housed in animal care facilities with a 12-h light/dark cycle and a temperature of 24°C. Rats also had unrestricted access to water and rat pellets (Gold Coin, Malaysia). The rat pellet has an approximate nitrogen-free extract content of 49%, a minimum crude protein content of 22%, a maximum crude fiber content of 5%, a minimum crude fat content of 3.0%, a maximum moisture content of 13%, and a minimum ash content of 8%. The bedding for the rats was Kenaff (Muhaaz Enterprise, Terengganu, Malaysia) and was changed twice a week. Animals were separated into three groups: 18 male young SD rats aged 3 months, 18 male adults aged 9 months, and 18 old males aged 21 months (Makpol et al., 2020). Each group was further divided into two groups, with Group 1 receiving 1 mL of distilled water as the control (n = 8) and Group 2 receiving 200 mg/kg/day of soil *Z. officinale* Roscoe extract (n = 10) daily for 3 months. Prior to receiving therapy, each rat was kept in a cage for a week in a Sealsafe^®^ Plus Rat IVC Green Line (TECHNIPLAST, Varese, Italy) for acclimatization. For each test carried out, the animals were tested according to their groupings, commencing with Group 1 and moving on to Group 2. The Universiti Kebangsaan Malaysia Animal Ethics Committee approved the experimental plan (UKMAEC Approval Number: BIOK/PP/2018/SUZANA/14-MAY/924-JUNE-2018-MAY-2020). Figure 1 shows the experimental design for this research. The animal numbers were selected based on a previous study (Makpol et al., 2020).

### 2.8 Urine collection

One milliliter of rat’s urine was collected *via* spontaneous urination into a clear plastic bag on day 0 and day 90 of treatment using a pipette tip. Prior to analysis, the urine was stored in a freezer set at −80°C.

### 2.9 Blood collection

Blood was withdrawn from each rat through the orbital sinus collection technique on day 0 and day 90 of treatment. After centrifuging the collected blood in the EDTA tube at 3,000 rpm for 10 min at 4°C, the plasma was stored in a—80°C freezer until analysis.

### 2.10 Euthanisation of animals

KTX agents with a mixture of ketamine, xylazine, and zoletil-50 (tiletamine and zolazepam) were used as anesthetics in this study due to their effectiveness, speed, minimal discomfort, anxiety, and distress. The KTX agents were injected intraperitoneally at a dose of 0.1 mL/250 g of body weight (BW). They were sacrificed by decapitation.

### 2.11 Collection of organs

On day 90, all rats were fasted overnight before being euthanized for necropsy analysis. Gastrocnemius muscles were obtained from the rats. The organs were cleaned in 90% normal saline to remove any adhering tissue before weighing. The organ weight was measured as soon as possible to prevent drying, and it’s relative to the animals’ body weight (BW) was analyzed.

### 2.12 MDA analysis

Analysis of MDA in rat plasma was conducted in accordance with a previous study (Hamezah et al., 2017). The reagent was prepared through mixing of 5 mM DNPH (2,4-dinitrophenyl benzine) (Sigma Aldrich, St. Louis, MO, United States), 2 M HCl (hydrochloric acid) (Sigma Aldrich, St. Louis, MO, United States), 35% perchloric acid (HClO_4_) (Merck, Germany), 1% sulfuric acid (H_2_SO_4_) and 1.3 M NaOH (natrium hydroxide, Merck, Germany). 12.5 µL TEP (1,1,3,3-tetraethoxypropane) (Sigma Aldrich, St. Louis, MO, United States) and 50 mL 1% sulfuric acid were combined and incubated overnight at 4 °C to produce mega stock solution (1 mM stock solution). For working standard (100 uM), 500 μL TEP (Mega stock) was added to 4.5 mL mili Q water. 380 mL acetonitrile (HPLC Grade, Merck, Germany), 620 mL mili Q and 2 mL of acetic acid (Merck, Germany) were used as the mobile phase. This mixture was filtered by a .45 µm filter membrane using a vacuum pump and sonicated for 20 min to de-gas. A series of standard concentrations (Table 2) was prepared. Briefly, 50 μL standard/sample was added to 200 μL 1.3 M NaOH. This mixture was incubated in a 60°C water bath for 1 h for alkaline hydrolysis of protein-bound followed by 5 min of a cooling period in ice. Thirty-five percent perchloric acid was added and further centrifuged at 10,000 g, 10 min, and 4°C to precipitate the protein. Three hundred microliter supernatant was transferred and added with 12.5 μL, 5 mM DNPH. This mixture was incubated at room temperature for 30 min. Standard/sample was filtered (size .45 µm) and transferred into an insert in the vial.

**TABLE 2:** Standard concentration for MDA analysis.

MDA (μM)	Mili Q H_2_0 (μL)	Working standard
10	900	100
20	800	200
30	700	300
40	600	400
50	500	500

Five microliter of sample was injected onto the UPLC system (Waters Alliance 2,695, United States) equipped with PhotoDiode Array Detector (Waters 2,996, United States). The reverse-phase Acquity UPLC^®^ BEH C18, 1.7 m, 2.1 mm 50 mm column was part of the UPLC system. Photodiode array detection was set at a wavelength of 310 nm and a programmed solvent delivery system with a flow rate of 0.4 mL/min (Waters Corporation, Milford, MA, United States). By contrasting the retention period of the sample with the established standard, plasma MDA was determined. The sample’s MDA had a 2.6-min retention period, which permitted an entire chromatographic run every 5 minutes. The peak area of the external standards was used to compute the MDA concentrations, and the results from the calibration curves were expressed in nmol/mL. Each sample was analyzed simultaneously.

### 2.13 Antioxidant enzyme activity

The activities of the antioxidant enzymes catalase (CAT) and superoxide dismutase (SOD) in rat plasma were assessed using Catalase Assay kit (Cayman, Ann Arbor, MI, United States) and Superoxide Dismutase Assay Kit (Cayman, Ann Arbor, MI, United States), by referring to the kits’ manuals. The absorbance was measured at wavelengths of 540 nm for CAT and 440–460 nm for SOD by EnSpire Multimode Plate Reader (Perkin Elmer, Singapore). The results were presented as μmol/min/mL and U/mL for CAT and SOD activity, respectively. A bsorbance was measured at wavelengths of 540 nm for CAT and 440–460 nm for SOD. The results were presented as μmol/min/mL for CAT activity. SOD activity was shown as U/mL.

### 2.14 Analysis of urinary oxidative stress

Urine samples from each rat were tested for 15-isoprostane F_2t_ activity using a urinary isoprostane ELISA kit (Oxford Biomedical Research, Oxford, United States) according to the manufacturer’s instruction. The sample was prepared by adding 100 L of urine to an anti-15-isoprostane F_2t_-coated well plate following dilution with glucuronidase. The 15-isoprostane F_2t_ horseradish peroxidase (HRP) conjugate and tetramethylbenzidine (TMB) substrate were then added to the mixture. An EnSpire Multimode Plate Reader (Perkin Elmer, Singapore) quantified the produced colour as the absorbance at 650 nm. Based on the data collected and the standard curve produced using the given standard solution, the isoprostane concentration (ng/mL) of each sample was calculated.

### 2.15 Analysis of creatine kinase-MM (CK-MM)

CK-MM activity was assessed using a Rat CK-MM ELISA Kit (Life Diagnostics Inc., West Chester, PA, United States) in accordance with the manufacturer’s instructions. The sample was prepared by diluting a total of 25 L of plasma with a diluent before being transferred to microtiter plates that were coated with an anti-rat CK-MM antibody. Then, TMB reagent was added to each well and lastly, enzyme conjugates. After adding the stop solution to halt the reaction, the plates were gently shaken so that their absorbance could be measured at 450 nm using an EnSpire Multimode Plate Reader (Perkin Elmer, Singapore). Based on the results, each sample’s CKMM concentration (ng/mL) was determined using the standard curve that was plotted using the supplied standard solution.

### 2.16 Analysis of skeletal muscle oxidative stress

Lipid peroxidation marker, and lipid hydroperoxides were analyzed using Lipid Hydroperoxide (LPO) assay kit (Cayman Chemical, Ann Harbor, MI, United States), in accordance with the manufacturer’s instructions. To prevent batch-to-batch variability, all samples for each lipid peroxidation marker were examined in the same batch. In summary, 100 mg of gastrocnemius muscle was homogenized using PBS buffer containing .5 M BHT. After adding the R2 reagent, clear supernatant homogenates were generated, which were then combined with the diluted R1 reagent. Through redox interactions with ferrous ions and thiocyanate as the chromogen, this kit directly assessed the lipid hydroperoxide radicals. Three duplicates of each sample were plated. By using an EnSpire Multimode Plate Reader (Perkin Elmer, Singapore) at 500 nm, the supernatant absorbance was measured. The data were computed to determine the concentration of each sample using a standard curve from the provided standard solution. By adjusting each sample’s spectrophotometrically measured absorbance (500 nm) to *μ*M using a hydroperoxide concentration standard curve, the average lipid hydroperoxide concentration for each sample was determined using this calculation:

Hydroperoxide concentration in sample (µM):

$$\frac{Hydroperoxide\;values\;of\;the\;sample\;tubes\;\left(HPST\right)}{Volume\;of\;extract\;used\;for\;the\;assay\;\left(VE\right)}\;X\;\frac{1\;mL}{Volume\;of\;the\;original\;sample\;used\;for\;extraction\;\left(SV\right)}$$

### 2.17 Measurement of inflammatory biomarkers

By using an ELISA kit from Elabscience Biotechnology (United States), the levels of inflammatory biomarkers including IL-6 (E-EL-R0015), TNF-α (E-EL-R0019), and CRP (E-EL-R3002) were assessed in a 96-well plate. In order to get the plasma, the blood was collected into heparin tubes and separated for 15 min at 1,000 g at 4°C. The 96-well plate was then filled with the plasma, which was then incubated for 90 min at 37°C. Utilizing the EnSpire Multimode Plate Reader (Perkin Elmer, Singapore), the absorbance of IL-6, TNF-α, and CRP was measured at 450 nm and compared to the standard curve.

### 2.18 Statistical analysis

The results of each experiment were recorded as mean ± standard deviation, with each experiment carried out in triplicate. One-way or two-way ANOVA was used to analyze the significant differences, and multiple comparisons were performed using the Bonferroni or Tukey test *post hoc* test. Levene’s test was used to test the homogeneity of the variances. Old rats and young rats were compared using Student's t-tests. *P* < .05 was considered as a significant difference in the analysis, which was conducted using SPSS software version 28.

## 3 Results

### 3.1 Determination of total yield of dry extract

**FIGURE 2:**
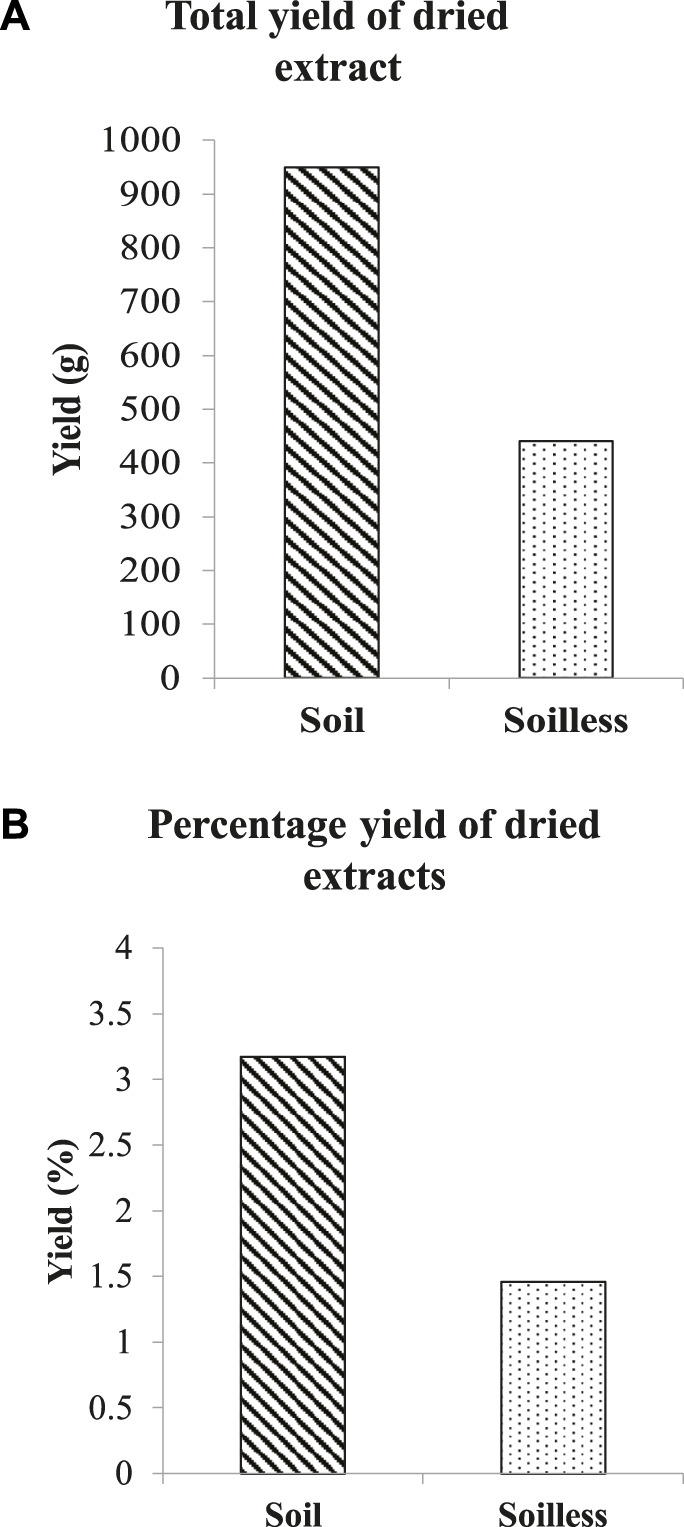
Total yield of dried extracts of soil and soilless ginger in g **(A)** and % **(B)**.

Thirty kilograms of soilless ginger yielded 440 g (1.46%) of ginger extract while 950 g (3.17%) of ginger extract was yielded from soil ginger (Figures 2A, B).

### 3.2 Total amount of phenolic compounds, [6]-Gingerol and [6]-Shogaol (µg/mL)

**FIGURE 3:**
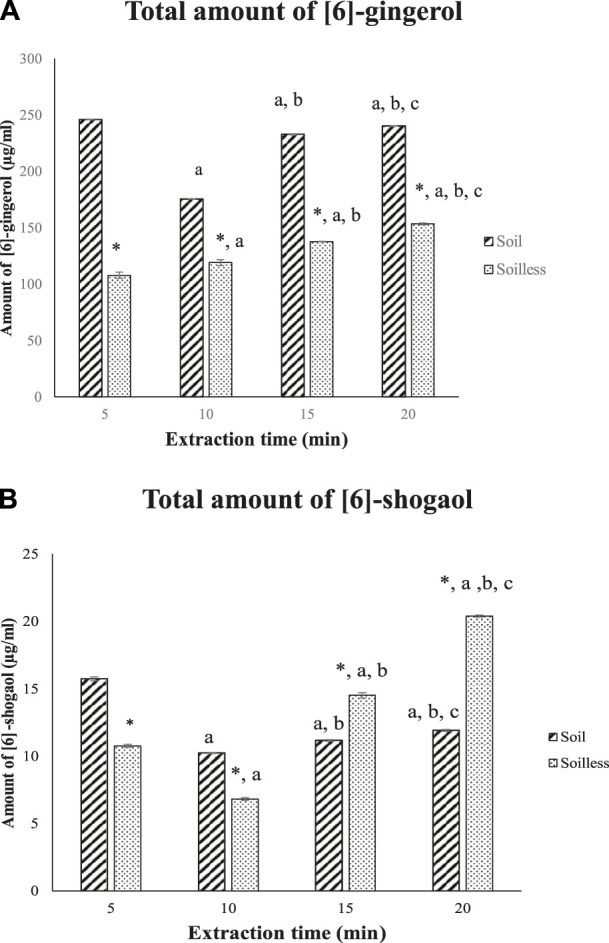
Total amount of [6]-gingerol **(A)** and [6]-shogaol **(B)** (µg/mL) for both soil and soilless ginger. Data presented as mean ± SD (n = 6), *p < .05 indicates a significant difference from the soil at the corresponding extraction time ^a^ *p* < .05 indicates a significant difference from the extraction time of 5 min; ^b^ *p*<.05 indicates a significant difference from the extraction time of 10 min; And ^c^ *p* < .05 indicates a significant difference from the extraction time of 15 min

In comparison to soilless ginger, the total amount of [6]-gingerol was significantly higher in soil ginger extract at 5, 10, 15, and 20 min of extraction (*p* < .05) (Figure 3A). On the other hand, the total amount of [6]-shogaol was higher in 5 min and 10 min of extraction time compared to soilless ginger (*p* < .05). Contrarily, compared to soil ginger, the amount of [6]-shogaol in soilless ginger was significantly increased with increasing extraction time (15 min and 20 min) (*p* < .05) (Figure 3B).

### 3.3 Total percentage of phenolic compounds, [6]-Gingerol and [6]-Shogaol (%)

**FIGURE 4:**
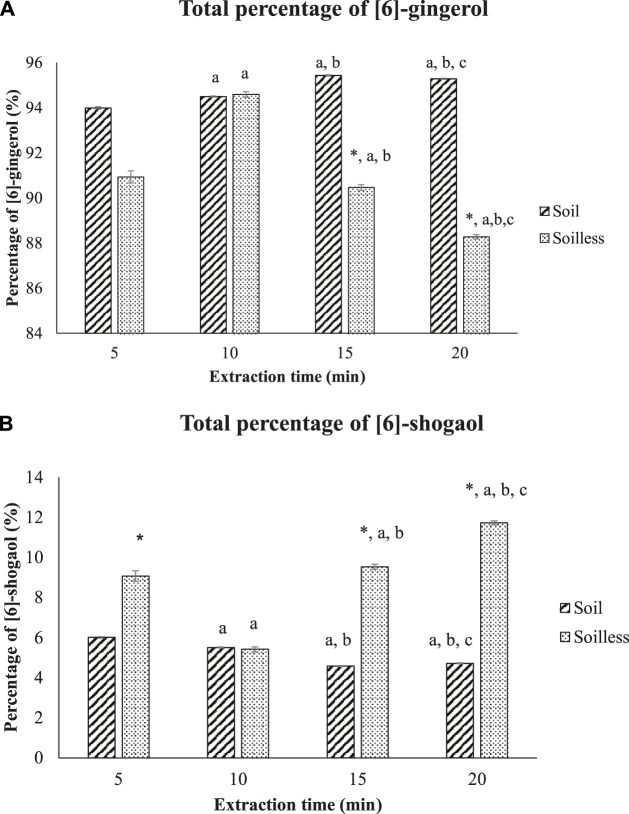
Percentage of [6]-gingerol **(A)** and [6]-shogaol **(B)** in both soil and soilless ginger. Data presented as mean ± SD (n = 6), *p < .05 indicates a significant difference from the soil at the indicated extraction time; ^a^ *p* < .05 indicates a significant difference from the extraction time of 5 min; ^b^ *p* < .05 indicates a significant difference from the extraction time of 10 min; ^c^ *p*<.05 indicates a significant difference from the extraction time of 15 min.

The total percentage of [6]-gingerol was significantly higher in soil ginger extract at 5, 15 and 20 min of extraction time compared to soilless ginger at respective extraction times. No significant difference in the percentage of [6]-gingerol was observed at 10 min of extraction time between soil and soilless ginger although the percentage of [6]-gingerol increased significantly during this interval compared to 5 min of extraction time (*p* < .05) (Figure 4A). In contrast, the percentage of [6]-shogaol was significantly higher in soilless ginger at 5, 15 and 20 min of extraction time (*p* < .05, Figure 4B) compared to soil ginger.

### 3.4 DPPH radical scavenging activity

**FIGURE 5:**
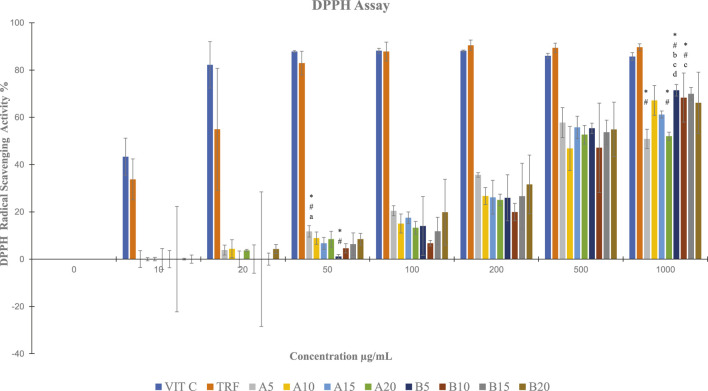
DPPH radical scavenging activity in both soil and soilless ginger. Data presented as mean ± SD (n = 3). A5, A10, A15 and A20 denote soilless ginger groups. B5, B10, B15, B20 denote soil ginger groups, *p < .05 significantly different from Vit C, ^#^ *p* < 005 significantly different from TRF, ^a^ *p* <.05 significantly different from soil ginger at the same extraction time, ^b^ *p* <.05 significantly different from soilless ginger at the same extraction time, ^c^ *p* <.05 significantly different from A5 at the same extraction time, and ^d^ *p* <0.05 significantly different from A20 at the same extraction time.

By using DPPH assay, soil ginger at 5 min of extraction time (B5) with a concentration of 1,000 μg/mL exhibited higher antioxidant activities than soilless ginger at 5 min and 20 min of extraction times (A5, A20) with similar concentrations (Table 3; Figure 5).

**TABLE 3:** DPPH radical scavenging activity in both soil and soilless ginger. Data presented as mean ± SD (n = 3).

Concentration µg/mL	% DPPH radical scavenging activity assay							
	0	10	20	50	100	200	500	1,000
Vit C	.00 ± 0	43.35 ± 7.8	82.22 ± 9.8	87.86 ± 0.4	88.20 ± 1.0	88.12 ± 0.4	86.06 ± 1.0	85.76 ± 1.6
TRF	.00 ± 0	33.75 ± 8.7	54.95 ± 25.8	82.95 ± 5.0	87.84 ± 4.0	90.51 ± 2.2	89.40 ± 2.0	89.68 ± 1.5
A5	.00 ± 0	.00 ± 3.6	3.86 ± 2.1	11.77 ± 2.4*^#^	20.48 ± 2.1	35.64 ± 1.0	57.79 ± 6.4	50.90 ± 4.1*^#^
A10	.00 ± 0	.00 ± 0.7	4.38 ± 3.8	8.92 ± 2.6	15.06 ± 4.0	26.70 ± 3.6	46.86 ± 9.3	67.19 ± 6.3
A15	.00 ± 0	.00 ± 0.7	.00 ± 3.4	6.69 ± 2.5	17.54 ± 2.5	26.18 ± 7.1	55.73 ± 4.8	61.18 ± 1.5
A20	0.00 ± 0	0.00 ± 4.45	3.67 ± 0.4	8.44 ± 3.36	13.29 ± 2.66	25.02 ± 2.45	52.66 ± 3.96	52.02 ± 1.75*^#^
B5	.00 ± 0	.00 ± 3.64	.00 ± 5.98	1.11 ± 0.91*^#a^	14.02 ± 12.45	25.96 ± 9.71	55.37 ± 2.18	71.46 ± 2.44*^#ac^
B10	0.00 ± 0	0.00 ± 22.3	0.00 ± 28.4	4.57 ± 1.96	6.71 ± 1.16	19.93 ± 3.67	47.15 ± 18.9	68.36 ± 10.4*^#b^
B15	.00 ± 0	.00 ± 0.37	.00 ± 2.58	6.31 ± 4.81	11.84 ± 5.87	26.68 ± 13.94	53.72 ± 5.06	69.98 ± 2.75
B20	.00 ± 0	.00 ± 1.76	4.28 ± 1.91	8.42 ± 2.46	19.86 ± 13.9	31.62 ± 12.4	54.90 ± 11.47	66.16 ± 12.9

A5, A10, A15 and A20 denote soilless ginger groups. B5, B10, B15, B20 denote soil ginger groups, *p < .05 significantly different compared to Vit C, #p < 005 significantly different compared to TRF, ap<.05 significantly different compared to soilless ginger at similar concentration, bp<.05 significantly different compared to A5 at similar concentration, cp<.05 significantly different compared to A20 at similar concentration.

### 3.5 FRAP activities

**FIGURE 6:**
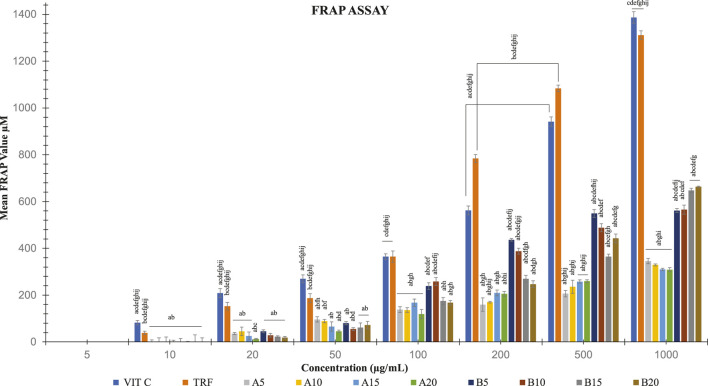
FRAP activities in both soil and soilless ginger. The data is shown as mean SD (*n* = 6). A5, A10, A15 and A20 denote soilless ginger groups. B5, B10, B15, B20 denote soil ginger groups. ^a^ *p*<0.05 is significantly different from TRF, ^b^ *p*<0.05 is significantly different from Vit C, ^c^ *p*<0.05 is significantly different from A5, ^d^ *p*<0.05 is significantly different from A10, ^e^ *p*<0.05 is significantly different from A15, ^f^ *p*<0.05 is significantly different from A20, and ^g^ *p*<0.05 is significantly different from B5, ^h^ *p*<0.05 is significantly different from B10, ^i^ *p*<0.05 is significantly different from B15 and ^j^ *p*<0.05 is significantly different from B20.

Soil ginger with a concentration of 1,000 μg/mL at all extraction times (B5, B10, B15 B20) exhibited higher antioxidant activities than the soilless ginger at similar concentrations and respective extraction times (A5, A10, A15, A20) with the highest activity was by soil ginger at 20 min of extraction time (B20) (Table 4; Figure 6).

**TABLE 4:** FRAP activities of soil and soilless ginger and controls. Data presented as mean ± SD (n = 6).

Mean FRAP value, µM								
Concentration µg/mL	0	10	20	50	100	200	500	1000
TRF	0.00 ± 0	38.81 ± 7.23	152.52 ± 16.64	187.33 ± 19.28	364.37 ± 24.50	783.63 ± 16.97	1083.63 ±13.58	1311.04 ± 18.10
VIT C	0.00 ± 0	82.52 ± 9.32	208.81 ± 18.90	269.93 ± 16.86	364.37 ± 12.49	562.15 ± 19.13	941.41 ± 20.53	1386.59 ± 24.70
A5	0.00 ± 0	0.00 ± 8.7	36.08 ± 4.33^abf^	96.36 ± 11.68^abfh^	138.31 ± 12.29	159.42 ± 28.66	206.36 ± 13.57	345.53 ± 11.25
A10	0.00 ± 0	0.00 ± 17.29	45.25 ± 18.28	88.86 ± 6.99^abf^	136.64 ± 9.94	170.25 ± 3.63	236.08 ± 28.00	329.14 ± 4.59
A15	0.00 ± 0	0.00 ± 20.97	25.63 ± 17.01	66.00 ± 19.75	168.22 ± 15.40	209.33 ± 12.62	257.85 ± 7.06	309.70 ± 5.25
A20	0.00 ± 0	0.00 ± 7.56	12.67 ± 2.94	46.00 ± 4.44^abd^	118.96 ± 19.76	205.63 ± 10.26	259.70 ± 6.32	308.96 ± 8.63
B5	0.00 ± 0	0.00 ±14.82	45.81 ± 5.91	80.25 ± 6.82	238.86 ±14.35^abcdef^	436.36 ± 5.36^abcdefij^	548.86 ± 16.86^abcdefhij^	561.36 ± 8.22^abcdefij^
B10	0.00 ± 0	0.00 ± 2.55	28.31 ± 8.01	54.97 ±6.68	258.31 ± 17.17^abcdefij^	387.47 ± 12.54^abcdefgij^	488.31 ± 17.68^abcdef^	565.53 ± 19.41^abcdef^
B15	0.00 ± 0	0.00 ± 30.75	22.67 ± 3.33	61.56 ± 20.16	174.89 ± 15.03	269.89 ± 14.37^abcdfgh^	364.33 ±10.93^abcefgh^	647.67 ± 8.82^abcdefg^
B20	0.00 ± 0	0.00 ± 17.66	18.78 ± 5.09	72.11 ± 16.02	167.67 ± 9.28	247.11 ± 14.37^abdgh^	442.67 ± 18.56^abcdefg^	663.22 ± 2.55^abcdefg^

A5, A10, A15 and A20 denote soilless ginger groups. B5, B10, B15, B20 denote soil ginger groups.

^a^ *p*<0.05 significantly different compared to TRF,

^b^ *p*<0.05 significantly different compared to Vit C,

^c^ *p*<0.05 significantly different compared to A5,

^d^ *p*<0.05 significantly different compared to A10,

^e^ *p*<0.05 significantly different compared to A15,

^f^ *p*<0.05 significantly different compared to A20,

^g^ *p*<0.05 significantly different compared to B5,

^h^ *p*<0.05 significantly different compared to B10,

^i^ *p*<0.05 significantly different compared to B15,

^j^ *p*<0.05 significantly different compared to B20.

### 3.6 Level of oxidative stress markers in the tissue, plasma, and urine

**FIGURE 7:**
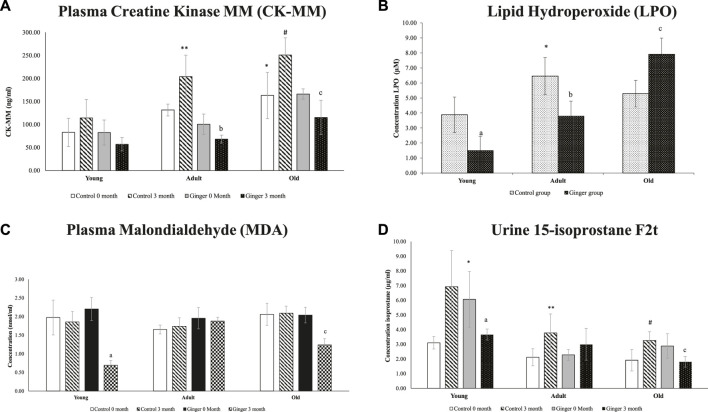
Plasma Creatine Kinase MM (CK-MM) **(A)**, lipid hydroxyperoxide **(B)**, plasma malondialdehyde (MDA) **(C)** and urine 15-isoprostane F2t **(D)** concentrations in young, adult, and old rats. The data were presented as mean ± SD, *p < .05 significantly different compared to young control rats at 0 month, **p < .05 significantly different compared to adult control rats at 0 month. #*p* < .05 significantly different compared to old control rats at 0 month, ^a^ *p*<.05 significantly different compared to young ginger rats at 0 month, ^b^ *p*<.05 significantly different compared to adult ginger rats at 0-month, ^c^ *p*<.05 significantly different compared to old ginger rats on at 0 month.

When compared to the young control rats at month 0, the plasma level of CKMM was significantly higher in the older rats (*p* < .05) (Figure 7A). At month 3, adult and elderly rats were shown to have similar increases in plasma CKMM compared to their respective control groups (*p* < .05). In comparison to the untreated adult and old rats at month 0, ginger administration significantly reduced plasma CKMM in both groups at month 3 (*p* < .05). For LPO, when compared to young control rats, lipid hydroxyperoxide was significantly higher in adult control rats (*p* < .05). When compared to the untreated groups, ginger treatment significantly decreased LPO levels in both young and adult rats (*p* < .05). As opposed to their untreated aged group, ginger therapy dramatically raises the LPO level in old rat groups. (*p* < .05) (Figure 7B). We observed a stable MDA level in all control rats. When compared to the untreated young and old rats at month 0, ginger treatment dramatically lowered the MDA levels in both young and old rats at 3 months of treatment. (*p* < .05) (Figure 7C). At month 0 compared to their controls, the urine 15-isoprostane F2t levels were significantly higher in the adult and old groups. Compared to the untreated groups at month 0, ginger treatment significantly reduced the concentration of 15-isoprostane F_2t_ in both young and old rat groups at month 3 (*p* < .05, Figure 7D).

### 3.7 Level of antioxidant enzymes

**FIGURE 8:**
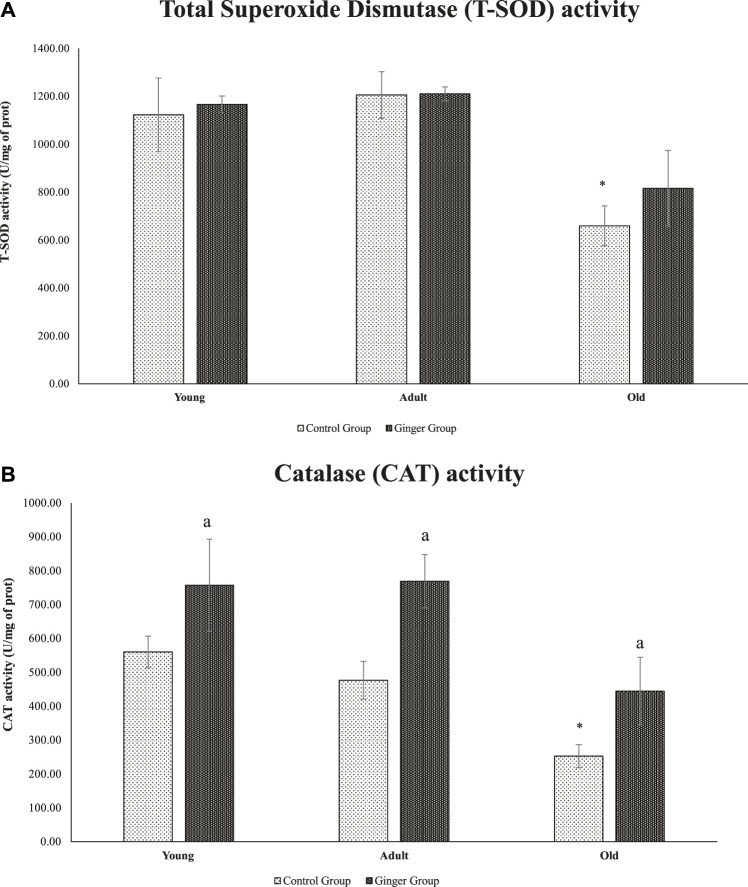
Age-related differences in T-SOD activity **(A)** and catalase activity of young, adult, and old rats **(B)**. The data was presented as mean ± SD, *p < .05 significantly different compared to young control rats, ^a^ *p*<.05 significantly different compared to untreated rats at respective months.

When compared to young control rats, there was a substantial decline in T-SOD activity (*p* < .05) in old rats. On the other hand, neither the ginger intervention nor the control significantly changed the SOD activity in young or adult rats (Figure 8A) after 3 months of treatment. In terms of catalase activity, when compared to young control rats, the catalase activity in old rats was significantly reduced (*p* < .05). Interestingly, rats of all ages treated with ginger had significantly higher catalase activity than the untreated rats (*p* < .05, Figure 8B).

### 3.8 Level of plasma pro-inflammatory biomarkers

**FIGURE 9:**
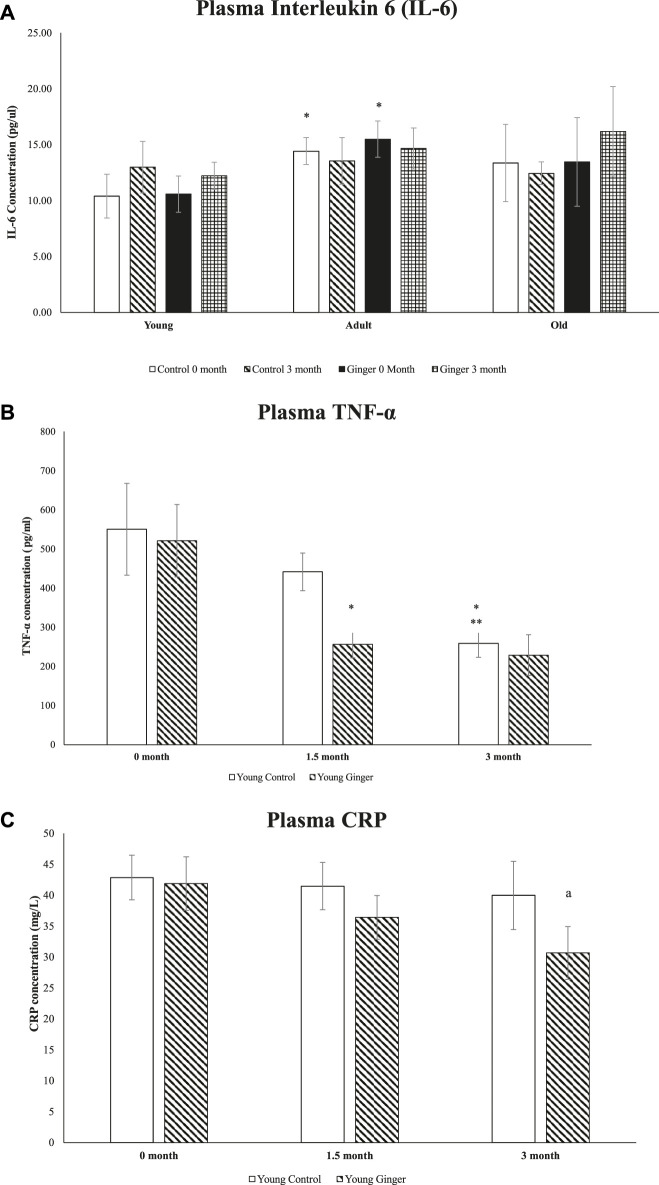
Plasma interleukin 6 (IL-6) levels in young, adult, and old rats (A), plasma levels of TNF-α **(B)** and CRP (C) in young rats. The data were presented as mean ± SD. ^*^ *p* < 0.05 significantly different compared to young control rat at 0 month, **p < 0.05 significantly different compared to young control rat at 1.5 months, ^a^ *p*<0.05 significantly different compared to untreated rats at respective months.

When compared to young control rats at 0 months, adult control and baseline untreated adult rats had significantly higher plasma IL-6 concentrations (*p* < .05, Figure 9A). For the whole course of treatment, there were no significant changes in plasma IL-6 concentration between young, adult and old rats. For TNF-α, when compared to the young control at 0-month and 1.5-month intervals, the young control’s plasma TNF-α level at 3 months was significantly decreased. Although the ginger treatment did not significantly reduce TNF-α concentration at months 0 and 3, it did significantly reduce TNF-α concentration in young, treated rats at intervals of 1.5 months when compared to the untreated group (*p* < .05, Figure 9B). On the other hand, young control groups’ plasma CRP levels remained constant at all intervals during the entire course of treatment. However, compared to the untreated group, the young rats treated with ginger extract for 3 months with ginger extract had lower plasma CRP levels (*p* < .05, Figure 9C).

## 4 Discussion

The use of natural sources of bioactive chemicals as nutraceuticals to support human health has drawn a lot of interest lately. The natural source of phenolic component used in this study was *Zingiber officinale* Roscoe. We used water for extraction since a number of scientific studies revealed that extraction technologies that primarily use organic solvents may leave an unwelcome hazardous residue in the finished product (Soquetta et al., 2018; Chaves et al., 2020; Carpentieri et al., 2021). Organic volatile solvents such as acetone, chloroform, ethyl ether, ethanol, dichloromethane, ethyl acetate, hexane, benzene, and toluene are highly toxic, human carcinogens, environmental hazards, non-genotoxic animal carcinogens. Accumulation and metabolism of the volatile solvent residue can generate highly toxic metabolites which bind covalently to some macromolecules and produce toxic effects (Opuni et al., 2021). Due to their high solubility in fat, the unmetabolized residual solvents accumulate in fatty tissues such as those of the nervous system which may cause neurotoxicity (Joshi and Adhikari, 2019).

Our results showed that soil ginger possessed a higher extraction yield than soilless ginger. Similar to this, a prior study found that soil-grown lettuce produced more and had leaves with a greater nitrate content than soilless lettuce (Fussy and Papenbrock, 2022). Nitrates in the soil are a primary source of nitrogen which is essential for plant growth. Fresh ginger typically contains between 85 and 95 percent water, making them vulnerable to microbial deterioration and chemical degradation (An et al., 2016). Volatile compounds make up 97 percent of the *Zingiber officinale* rhizome’s constituents in essential oils (Ghasemzadeh et al., 2018). The primary source for ginger’s bioactivities came from its rhizomes which consist of non-volatile substances (oleoresins). A previous study reported the most significant categories among these are gingerols, shogaols, and paradols (Arcusa et al., 2022).

According to the outcomes of the ultrahigh-performance liquid chromatography (UHPLC), the active components in our extract were discovered to be [6]-gingerol and [6]-shogaol (0.6% w/w). The main polyphenols in fresh ginger are [6]-gingerols, however with heat treatment or prolonged storage, gingerols can change into their corresponding [6]-shogaols (An et al., 2016). Among the factors influencing this transformation are acidity, temperature, and the length of the extraction (Ghasemzadeh et al., 2018). When [6]-gingerols are converted into [6]-shogaols, it takes place in an acidic aquatic environment (Ghasemzadeh et al., 2018). The acidic solutions facilitate the dehydration of 6-gingerol to produce 6-shogaol. In aqueous solutions, these acids form different anions which can catalyze the conversion of gingerols to the corresponding shogaols. In line with this, we observed an increased level of [6]-shogaols with increased of extraction time in soilless ginger. A similar increase in other extracted phytochemicals was observed using soilless culture compared to soil-based plants (Verdoliva et al., 2021). Crop production and quality in soil and soilless systems depend on how well the plant absorbs nutrients from the growing medium, which is impacted by the number of nutrients present in the medium, the sources of the nutrients, or interactions between the different nutrients (Sambo et al., 2019). Active components in the material, which may disintegrate at high temperatures, can be preserved by drying them at a low temperature. However, it has been recommended that ginger be heated to increase its curative properties (Jung et al., 2018).

An earlier investigation revealed that ginger’s total phenolic content was linearly correlated with antioxidant activity (Ghasemzadeh et al., 2018). Prior work found that providing ginger to old animals successfully reduced DNA oxidative damage (Makpol et al., 2020). We supported this observation with our DPPH scavenging activity assays and FRAP activity assays. The organic nitrogen free radical known as the DPPH free radical has a deep purple colour which turned yellow during the test. Antioxidants contained in the ginger extract donate hydrogen in order to scavenge the DPPH free radical in the DPPH assay, which helped to create the non-radical form of DPPH (Mohd Sahardi et al., 2021). In FRAP assay, the identified antioxidant property changed the ferrous ion (Fe^2+^) from the ferric ion (Fe^3+^), causing a blue complex (Fe^2+^/TPTZ) to form (Spiegel et al., 2020). This is because antioxidants are reducing agents, which means they can aid in the reduction process by providing one electron or one hydrogen. In comparison to soilless ginger, we observed an increase in FRAP and DPPH radical scavenging activity with higher ginger extract concentrations and longer extraction periods. This increased antioxidant power can be due to the soil ginger’s higher yield of total [6]-gingerols and [6]-shogaols, which resulted in a higher presence of total α, and β-unsaturated ketones moieties than in soilless ginger. In comparison to *Moringa oleifera*, Kelulut honey (Mohd Sahardi et al., 2021), and other spices extracts (Abdul Qadir et al., 2017), ginger showed stronger antioxidant activity.

ROS are continuously produced *via* cell metabolism in trace amounts. However, excessive ROS production damages macromolecules and harms cells. Biomembranes can be destroyed by polyunsaturated fatty acids (PUFAs) that are esterified in membranes or store lipids due to ROS-induced peroxidation. It is produced from the peroxidation of arachidonic acid by free radicals without the aid of cyclooxygenase enzymes (Soffler et al., 2010). Ginger might reduce oxidative stress in ageing and diseased animal models. The increase in lipid peroxidation (LPO) that we saw in aged animals was also observed in pathological rat brains as reported by Sharma and Singh (2012). This study proposed that ginger ameliorated the condition, with a higher level of antioxidant enzyme activities including catalase observed, similar to our findings (Sharma and Singh, 2012). Numerous age-related disorders are linked to older individuals’ higher levels of lipid peroxide and malondialdehyde (MDA) end products and lower levels of antioxidants (Ali et al., 2022). LPO has been linked to the onset and progression of atherosclerotic illnesses, heart failure, and other cardiovascular disorders, according to several studies (Gianazza et al., 2020; Miyazawa, 2021). Measuring urine F_2t_ isoprostanes is one of the most accurate indicators of oxidative stress in living organisms (Guerreiro et al., 2015). Although they are stable, they have distinct half-lives in the blood (minutes) and urine (hours) (Turnbull et al., 2017).

In practically all organs, including skeletal muscle, oxidative damage increases with ageing. Aged rats have been demonstrated to develop sarcopenia phenotype as a result of macromolecule oxidative damage (Makpol et al., 2020). The signaling pathways that control the protein synthesis and proteolysis in muscle have been reported to be impacted by the imbalance of the redox state (Di Filippo et al., 2016). Because of this, oxidative stress indicators for ageing such as creatine kinase (CK) have been utilized. Creatinine, a breakdown product of the muscle’s creatine phosphate has been used as a measure of age-related decline in muscle mass. There are three isoenzymes of creatine kinase (CK), known as creatine phosphokinase: CK1, CK2, and CK3. Skeletal muscle contains the isoenzyme CK3, which comprises MM subunits (Mourad, 2021). The CK-MM released into the bloodstream reflects the integrity, stability, and function of the plasma membrane as well as the manifestation of mechanical and metabolic abnormalities inside the sarcomere of skeletal muscle (Kumar and Gill, 2018).

In this investigation, urine isoprostane F_2t_, LPO, and MDA levels were significantly higher in untreated old rats. However, in both young and old rats, CK-MM, LPO, and MDA levels were shown to be significantly lower after ginger administration. The antioxidant qualities of ginger may account for this finding. According to other *in-vivo* research, ginger’s active ingredients can boost antioxidant defense mechanisms including glutathione peroxidase and glutathione S-transferase, as well as lower the levels of malondialdehyde (MDA) and hepatic steatosis (Rahimlou et al., 2016). Through the activation of nuclear factor erythroid 2 (NFE2)-related factor 2 (Nrf2) and the expression of various antioxidant enzymes, ginger supplementation strengthens the antioxidant defense system and may therefore balance the redox state manifested by decrease in lipid peroxidation products.

We looked at the effects of ginger extract on the activity of antioxidant enzymes such as superoxide dismutase (SOD) and catalase to determine whether the Nrf2-inducing action of the ginger extract is connected to its capacity to induce endogenous antioxidant enzymes (CAT). SOD and CAT, a hemoprotein that lowers H_2_O_2_ and protects tissue from highly reactive OH• radicals catalyze the dismutation of superoxide radicals. They are regarded as major enzymes because they directly eliminate reactive oxygen species and guard cytosolic and membrane components from free radical damage. In young, adult, and old ginger-treated rats, ginger increased catalase’s catalytic activity, while we noticed decreased SOD activity in the old rats. This was consistent with earlier research that reported ginger extract has potent antioxidant activities and can serve a similar purpose as antioxidant enzymes including catalase (CAT), glutathione peroxidase (GPx), and superoxide dismutase (SOD) (Rostamkhani et al., 2022). Ginger’s anti-inflammatory characteristics benefited type 2 diabetics with insulin resistance by improving glucose tolerance and uptake in the body, which led to a reduction in insulin resistance (Pakan et al., 2021). Previous research had also suggested that [6]-shogaol modified KEAP1, a redox sensor and prevented Nrf2 from being degraded by proteasomes. Under dormant conditions, the Nrf2 is associated with its Kelch-like ECH-associated protein 1 (KEAP1) by forming the Nrf2-KEAP1 complex. Different alterations in KEAP1 structure induced by ROS lead to its dissociation from this complex and activation of Nrf2. The expression of Nrf2 target genes rises as a result of Nrf2 being translocated into the nucleus. GSH and glutathione levels rise as a result, and ROS levels drop (Mao et al., 2019). By altering proteins and genes, the extra ROS created has the potential to start and advance inflammatory disease pathways.

Immune cells produce a variety of cytokines and chemokines during inflammation in order to draw additional immune cells to the area of oxidative stress or infection (Chatterjee, 2016). In this work, we explored the potential effects of ginger supplementation (200 mg/day) on reducing proinflammatory biomarkers in rats. TNF-α, IL-6, and CRP were the three plasma proinflammatory biomarkers that were chosen as inflammatory state indicators. Interleukin-6 (IL-6) is known as the cytokine of the gerontologist (Rea et al., 2018). Our findings demonstrated that adult rats had levels of IL-6 that were noticeably higher than those of young rats. In addition to being able to create particular cellular and humoral immune responses, such as end-stage B cell differentiation, immunoglobulin production, and T cell activation. IL-6 also plays a significant role in the transition between acute and chronic inflammation (Yousif et al., 2021). Through local leukocyte recruitment, death, and migration, it stimulates an immunological response that can quickly eradicate the harmful substance (Choy and Rose-John, 2017). Age-related mild increases in circulating IL-6 levels are known, and their excessive presence in blood serum is a risk factor and potential biomarker for a variety of inflammatory disorders (Pogue et al., 2017). The results of our study’s IL-6 measurements agreed with those of Song et al. (2021). It demonstrated a general decrease in IL-6 levels following ginger supplementation, though it was not statistically significant (Song et al., 2021). It is likely that the ginger dosage and/or supplementation period were not sufficient to significantly reduce the inflammatory cytokines.

TNF-α and IL-6 may be in a connection that is susceptible to negative feedback. These biomarkers have a direct correlation, whereby an increase in one causes a decrease in the other and *vice versa*, according to a study conducted on mice (Yimin and Kohanawa, 2006). It is still unknown how exactly this interaction works and how TNF-α and IL-6 play different roles. In this study, treatment with ginger significantly lowered plasma levels of TNF-α at 1.5 months and CRP at 3 months in young rats, supporting this observation. A previous study reported lower mRNA expression of TNF-α in the soleus muscle of physically active mice following supplementation of black ginger extract for 4 weeks (Toda et al., 2016). It is possible that ginger’s anti-inflammatory properties could improve a person’s health acutely. This validates earlier research’s findings that ginger supplementation may be able to prevent the COX-2 inflammatory pathway, which raises oxidative stress and unfavorable cytokine inflammation (Fajrin et al., 2021). In addition to decreasing NF-κB activity, [6]-gingerol also inhibits the production of TNF-α, cyclooxygenase 2, and the by-products it generates, such as PGE2. As a result, acute-phase proteins like CRP are blocked in this process, which is consistent with the findings reported previously (Mazidi et al., 2016). Randomized clinical trials corroborated ginger’s action in TNF-α reduction (Rahimlou et al., 2016). Our findings support the claims made by other researchers that the consumption of ginger significantly lowers serum of CRP and TNF-α but not IL-6 (Jalali et al., 2020; Morvaridzadeh et al., 2020). Thus, we propose ginger as a nutraceutical agent in controlling inflammatory disorders due to its clinically substantial lowering CRP and TNF-α levels activities.

## 5 Conclusion

High biological activity in the soil and soilless ginger extracts is possible with sub-critical water extraction without hazardous solvents. Based on the current study’s findings, we confirmed that the SWE of both soil and soilless ginger possessed comparable antioxidant properties.

## 6 Recommendation

Further study is required to assess TNF-α and CRP levels in old rats since we did not measure them in this study design. However, soil ginger produced a higher yield of extracts with a more prominent antioxidant activity. The SWE of soil ginger treatment on the different ages of SD rats ameliorates oxidative stress and inflammation responses. This could serve as the basis for developing a nutraceutical agent that can be used as a therapeutic intervention for ageing-related diseases while minimizing the dangers associated with toxicological waste that will affect the environment in addition to being safe for human health. More data will be required from future research in order to establish clinical results, efficacy, and safety in a clinical context, as well as to understand the pathway modification of these polyphenols in reducing oxidative stress and their inflammatory response.

## Data Availability

The raw data supporting the conclusion of this article will be made available by the authors, without undue reservation.
